# Fuzheng–Buyi formula for treating castration-resistant prostate cancer: A systematic review and meta-analysis

**DOI:** 10.1097/MD.0000000000040275

**Published:** 2024-11-08

**Authors:** Xiao Liang, Shirong Peng, Hai Huang, Jia Xu, Wenjuan Liu, Hailong Wang, Qi Li

**Affiliations:** a Xiyuan Hospital, China Academy of Chinese Medical Sciences, Beijing, China; b Department of Urology, Sun Yat-sen Memorial Hospital, Sun Yat-sen University, Guangzhou, China.

**Keywords:** CRPC, Fuzheng–Buyi, meta-analysis, safety

## Abstract

**Background:**

The study was designed to systematically evaluate the efficacy and safety of Fuzheng–Buyi formula in treating castration-resistant prostate cancer (CRPC).

**Methods:**

A computer-based search were conducted in the databases, including CNKI, WanFang Data, VIP, CBM, PubMed, EMbase, and the Cochrane database to identify all randomized controlled trials. The studies investigating the efficacy and safety of Fuzheng–Buyi formula combined with Western medicine for the treatment of CRPC were included from January 1st, 2010 to December 31st, 2023. The quality of the included studies was evaluated according to the Cochrane Handbook manual, and meta-analysis was performed using Review Manager 5.3 and R Studio 4.2.3 software.

**Results:**

In this study, a total of 18 trials were included, encompassing a population of 1093 patients diagnosed with CRPC. The results of the meta-analysis showed that the combination of Fuzheng–Buyi formula and Western drugs was more effective in increasing the overall efficacy rate (risk ratio = 1.31, 95% confidence interval [CI] [1.17, 1.46], *P* < .00001), decreasing Traditional Chinese Medicine syndrome score (mean difference [MD] = −4.40, 95% CI [−6.10, −2.70], *P* < .00001), and quality of life scale (physiological condition MD = −2.31, 95% CI [−3.13, −1.48], *P* < .00001; social well-being MD = 1.26, 95% CI [0. 59, 1.94], *P* = .0002; emotional well-being MD = −2.04, 95% CI [−2.96, −1.12], *P* < .00001; functional well-being MD = −3.18, 95% CI [2.11, 4.26], *P* < .00001; others should be paid to MD = −3.15, 95% CI [−4.93, −1.37], *P* = .0005) compared with the Western medicine alone. And the incidence of adverse events was significantly lower in the combination treatment group compared with Western medicine group (risk ratio = 0.58, 95% CI [0.46, 0.73], *P* < .00001).

**Conclusion:**

The combination of Fuzheng–Buyi formula and Western medicine was more effective in improving the clinical efficacy and quality of life of CRPC patients, with lower incidence of adverse events compared with Western medicine alone.

## 1. Introduction

Prostate cancer is a prevalent malignancy of the male urinary tract, representing 27% of new malignant tumor cases in men worldwide and ranking first according to the Global Cancer Statistics Report 2022.^[1]^ In China, the incidence of prostate cancer is rapidly increasing, with an accompanying trend towards an earlier age of onset.^[2]^ Unfortunately, many patients with prostate cancer are diagnosed with advanced stages of the disease, including castration-resistant prostate cancer (CRPC), which has a poor prognosis and limited treatment options.^[3–5]^ Modern Western medicine has adopted endocrine therapeutic drugs, immunotherapeutic agents, and radiotherapy for the treatment of CRPC, however, the rapid resistance and adverse effects limited the application of those drugs.^[6–8]^ In Traditional Chinese Medicine (TCM) theory, the general pathogenesis of CRPC involves a combination of deficiency and excess, primarily attributed to an imbalance of yin and yang, insufficiency of kidney qi, deficiency of kidney yang, and depletion of yin essence. Treatment strategies based on the principles of tonifying kidney deficiency and eliminating pathogenic factors are recommended, which is a method of Fuzheng–Buyi.^[9–11]^ TCM may offer a complementary approach, with a wider range of targets and fewer side effects. The study was conducted to evaluate the efficacy and safety of Fuzheng–Buyi formula combined with Western medicine compared to conventional Western medicine in the treatment of CRPC.

## 2. Data and methods

This review adhered to the criteria outlined in the Preferred Reporting Items for Systematic Reviews and Meta-Analyses (PRISMA) guidelines, ensuring transparency and methodological rigor. The study protocol was preregistered on the International Prospective Register of Systematic Reviews (PROSPERO, CRD42023423352).

### 2.1. Inclusion criteria

The inclusion and exclusion criteria were developed in accordance with the PICOS principles of clinical trials, as described below:

1. Study population (P): patients with a clear diagnosis of prostate cancer by pathological examination and meeting the 2 diagnostic criteria of CRPC: serum testosterone at depleted levels (<50 ng/dL or < 1.7 nmol/L); 3 consecutive elevations of prostate-specific antigen (PSA) after a 1-week interval with a 50% or more increase in the lowest value.
2. Interventions and comparison groups: the treatment group consisted of individuals receiving Chinese herbal formulas with the effects of Fuzheng and Buyi in conjunction with conventional Western medicine. The Chinese herbal formulas utilized in this group were identified by names containing terms such as “Fuzheng,” “Yiqi,” “Buyi,” or were primarily composed of ingredients associated with Fuzheng and Buyi principles. In contrast, the control group solely received Western medicine. The Western medicine interventions encompassed endocrine therapy (ET), chemotherapy (CT), and other relevant measures, without containing any Chinese herbal ingredients or extracts.
3. Outcomes: (a) total effective rate (combined effective and ineffective grades)^[12]^; (b) TCM syndrome score^[13]^ is based on the assessment of patients’ symptoms before and after treatment, such as difficulty in urination, hematuria, painful urination, weakness, tongue coating, and pulse, etc. The degree is set into 4 grades (0–3) from none to severe, and the total is used to determine the evidence score; (c) functional assessment of cancer therapy-prostate^[14]^ is an assessment of prostate cancer patients’ physical, social/family, emotional, functional, and additional concerns, with 5 levels (0–4), where higher scores of physical, emotional, and additional concerns represent poor quality of life, while the remaining 2 items are the opposite; (d) incidence of adverse reactions including gastrointestinal reactions, bone marrow suppression, vomiting and nausea, anemia, etc; (e) PSA levels before and after treatment were compared between the 2 groups, and the efficacy was determined according to the change in values.
4. Study type (S): all relevant randomized controlled trials of Fuzheng–Buyi combined with Western medicine treatment versus Western medicine alone for CRPC.

### 2.2. Exclusion criteria

(1) Studies exhibiting substantial overlap in content or data, indicating an apparent reporting from the same study population, were excluded. (2) Studies presenting evident errors, inconsistencies, or suspected instances of plagiarism in the literature data were excluded. (3) Studies with incomplete information, where results could not be extracted, and important data could not be obtained by contacting the original authors, were excluded.

### 2.3. Search strategy

The study conducted a comprehensive literature search for randomized controlled trials on Fuzheng–Buyi formula in combination with western drugs for CRPC. The search was performed using mesh and entry terms on several databases including CNKI, WanFang Data, VIP, CBM, PubMed, EMbase, and the Cochrane Library from January 1st, 2010 to December 31st, 2023. To ensure the comprehensiveness of the literature search, a retrospective approach was adopted, whereby references from relevant articles were also included to address any potential gaps. The search terms included “destructive resistant prostate cancer,” “hormone-refractory prostate cancer,” “fuzheng,” “tonic,” “buyi,” “Quxie,” “traditional Chinese and Western medicine,” and “randomized controlled trial.”

### 2.4. Quality assessment and data extraction

The literature retrieved was imported into the EndNote X9.0.0 Literature Manager for checking and removing duplicates. Subsequently, the initial screening was conducted by reviewing the title and abstract of the text. The literature that met the exclusion criteria was then read in full to determine if it should be included. Two literature assessors independently performed the above work, and their results were compared. If necessary, third-party assessment decisions were requested. The information related to the included literature was also extracted and processed.

### 2.5. Evaluation of risk-of-bias of included studies

The methodological quality of the included studies was assessed by 2 reviewers according to the Cochrane Handbook for Systematic Reviews of Interventions Version 7.3. The assessment was conducted using R Studio 4.2.3 software and followed the 7 domains recommended by the Cochrane Handbook, which include random sequence generation, allocation concealment, blinding of participants and personnel, blinding of outcome assessment, incomplete outcome data, selective outcome reporting, and other sources of bias.

### 2.6. Meta-analysis

The statistical analysis of efficacy and safety indicators from the included studies was conducted using RevMan 5.3 software. The effect of continuous variables was evaluated using mean difference (MD), while the effect of dichotomous variables was evaluated using relative risk ratio (RR), and a 95% confidence interval (CI) was provided for both data. Statistical significance between the 2 groups was determined, and the *χ*^2^ test combined with I^2^ was used to assess the level of heterogeneity among the results of the studies. If *P* > .05 and I^2^ ≤ 50%, there was no statistical heterogeneity among the results, and a fixed-effects model was applied for the meta-analysis. If *P* ≤ .05 or *I*^2^ > 50%, it indicated significant heterogeneity among the studies, and subgroup analysis or sensitivity analysis was necessary to identify the source of heterogeneity. For this study, subgroup analysis was performed for the same outcome indicator with 10 or more included studies and heterogeneity present. For larger heterogeneity, sensitivity analysis was conducted using the one-by-one exclusion method, and the random-effects model was used for outcome analysis.

## 3. Results

### 3.1. Basic characteristics of included studies

**Figure 1.**
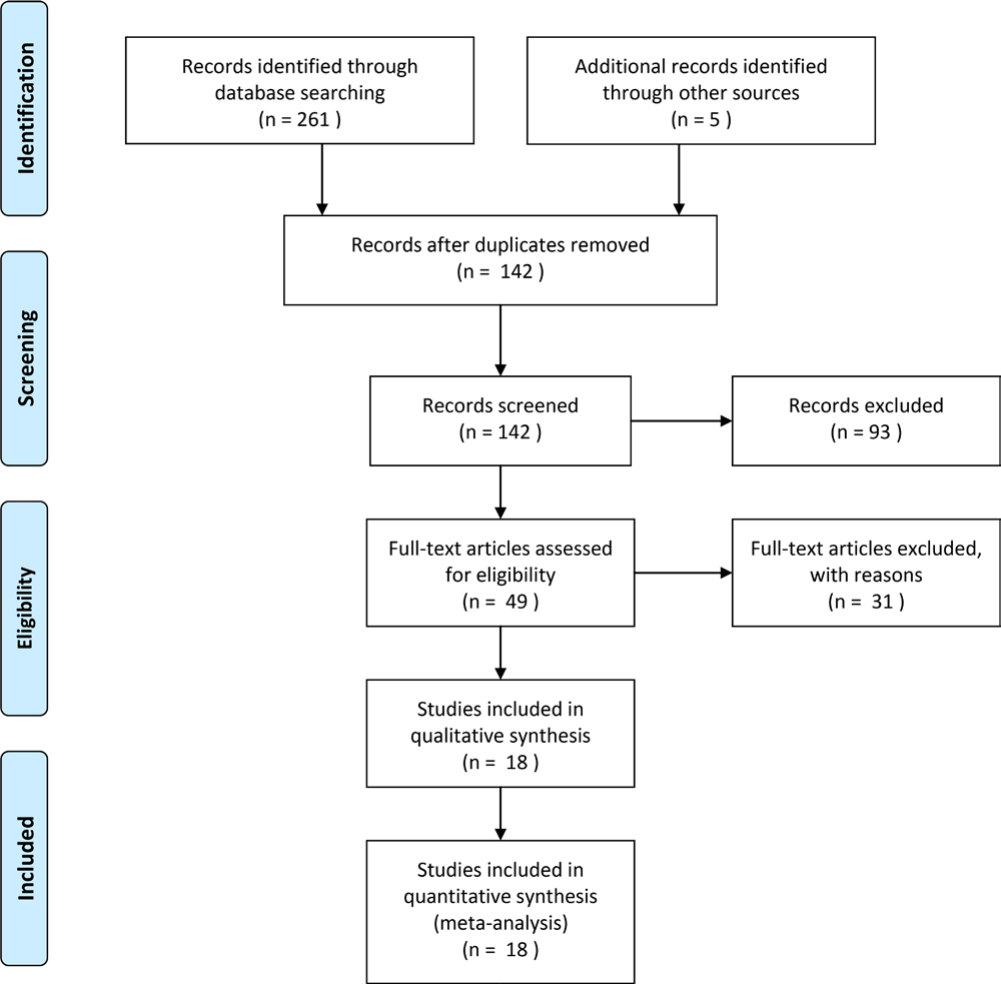
Flowchart of study setting. * The databases searched and the number of documents detected are specified as follows: CNKI (n = 26), WanFang Data (n = 43), VIP (n = 31), CBM (n = 23), PubMed (n = 72), EMbase (n = 65), and the Cochrane Library (n = 1).

A total of 266 relevant studies, all in Chinese, were obtained by database search and reference tracing. After de-duplication using the literature management software EndNote, 142 studies were obtained. After screening the titles and abstracts to eliminate reviews and conference reports, as well as animal experiments, 49 valid studies were obtained. The 49 articles were subsequently reviewed in full text. Among them, 25 studies did not combine Chinese and Western interventions, 3 studies were missing important information such as the exclusion standard or the basic information of patients was incomplete, and 3 studies failed to extract the main outcome indicators. Eighteen studies were finally included.^[15–32]^ The search process is shown in Figure 1. The basic characteristics of the literature are shown in Table 1.

**Table 1:** Study characteristics.

Author	Year	Country	Sample size (T/C)	Age (T/C)	Intervention		Duration (months)	Outcome
					T	C		
Wang WY et al^[15]^	2022	China	46 (23/23)	70.21 ± 8.78/68.16 ± 8.34	Compound KuShen injection + control	CT + ET	4	⑤
Zhang XQ et al^[16]^	2022	China	62 (33/29)	73.97 ± 6.10/72.48 ± 6.09	Jianpi Lishi Huayu formula + control	ET	1.5	①②③④
Zhang Y et al^[17]^	2020	China	56 (27/29)	72.5 ± 4.2/75.7 ± 5.1	CFF-1 + control	CT + ET	6	②③⑤
Mou RY et al^[18]^	2022	China	60 (30/30)	65.01 ± 10.16/64.52 ± 10.14	Jianpi Lishi Huayu formula + control	CT + ET	6	③
Wang W et al^[19]^	2021	China	63 (31/32)	64.53 ± 7.12/64.36 ± 7.07	Gushen Qushi Huazhuo decoction + control	ET	3	①②④⑤
Wang YQ et al^[20]^	2021	China	32 (16/16)	(60–65)/(60–65)	Yiqi Sanjie method + control	CT	1.5	④
Tang RZ et al^[21]^	2021	China	36 (18/18)	63.81 ± 5.27/63.29 ± 5.07	Yiai decoction + control	CT	1.5	①④⑤
Sun BY et al^[22]^	2020	China	116 (58/58)	64.11 ± 10.61/65.00 ± 10.09	Jianpi Lishi Huayu formula + control	CT + ET	2	②
Chen L et al^[23]^	2019	China	51 (26/25)	64.5 ± 13.1/64.8 ± 12.6	Zhou Qiling Tang + control	ET	2	①⑤
Gu DW et al^[24]^	2018	China	115 (57/58)	66.59 ± 1.48/68.55 ± 1.47	Qianlie Xiaozheng decoction + control	ET	3	⑤
Lu ZJ et al^[25]^	2018	China	72 (36/36)	73.9 ± 6.9/73.5 ± 7.1	Tonifying lung and kidney combined with resolving Stasis and detoxification + control	ET	3	②③⑤
Zhang Y et al^[26]^	2017	China	50 (24/26)	72.7 ± 7.1/71.2 ± 6.8	Fuyang Huayu prescription + control	ET	6	②③⑤
Feng SW^[27]^	2016	China	40 (20/20)	73.43 ± 5.25/72.85 ± 5.16	Chuanlongyiai decoction + control	ET	3	①④⑤
Kang CJ^[28]^	2015	China	60 (30/30)	(58–78)/(59–80)	Chuanlongyiai decoction + control	ET	3	①⑤
Chen YL et al^[29]^	2014	China	78 (39/39)	63.6 ± 3.2	Bushen Yiqi decoction + control	CT	2	①④
Pang R et al^[30]^	2013	China	63 (33/30)	72 ± 6/71 ± 6	Qianlie Xiaozheng decoction + control	ET	3	③⑤
Liu CW et al^[31]^	2011	China	58 (30/28)	(53–75)/(55–79)	Brucea javanica oil injection + control	CT	3	①④
Gu ZM et al^[32]^	2010	China	35 (19/16)	(57–88)/(55–89)	Fu zheng yi liu method + control	ET	3	①

C = control group; CT = chemotherapy; ET = endocrine therapy; T = treatment group; ① total effective rates; ② Traditional Chinese Medicine symptom scores; ③ functional assessment of cancer therapy-prostate scores; ④ adverse reaction; ⑤ prostate-specific antigen.

### 3.2. Evaluation of the quality of enrolled the literature

**Figure 2.**
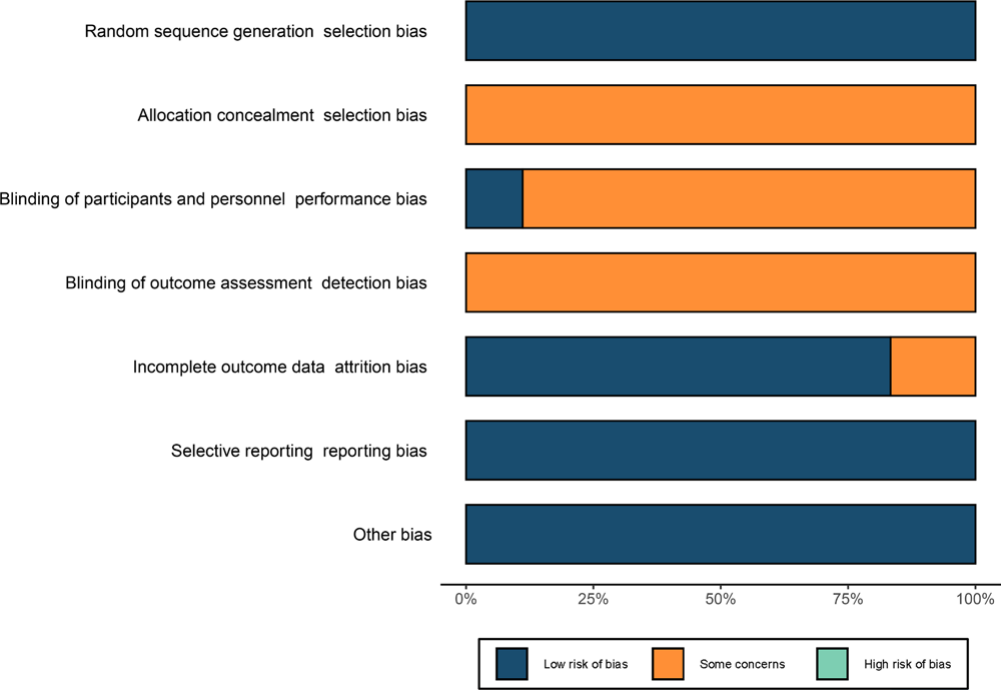
Risk offset ratio.

**Figure 3.**
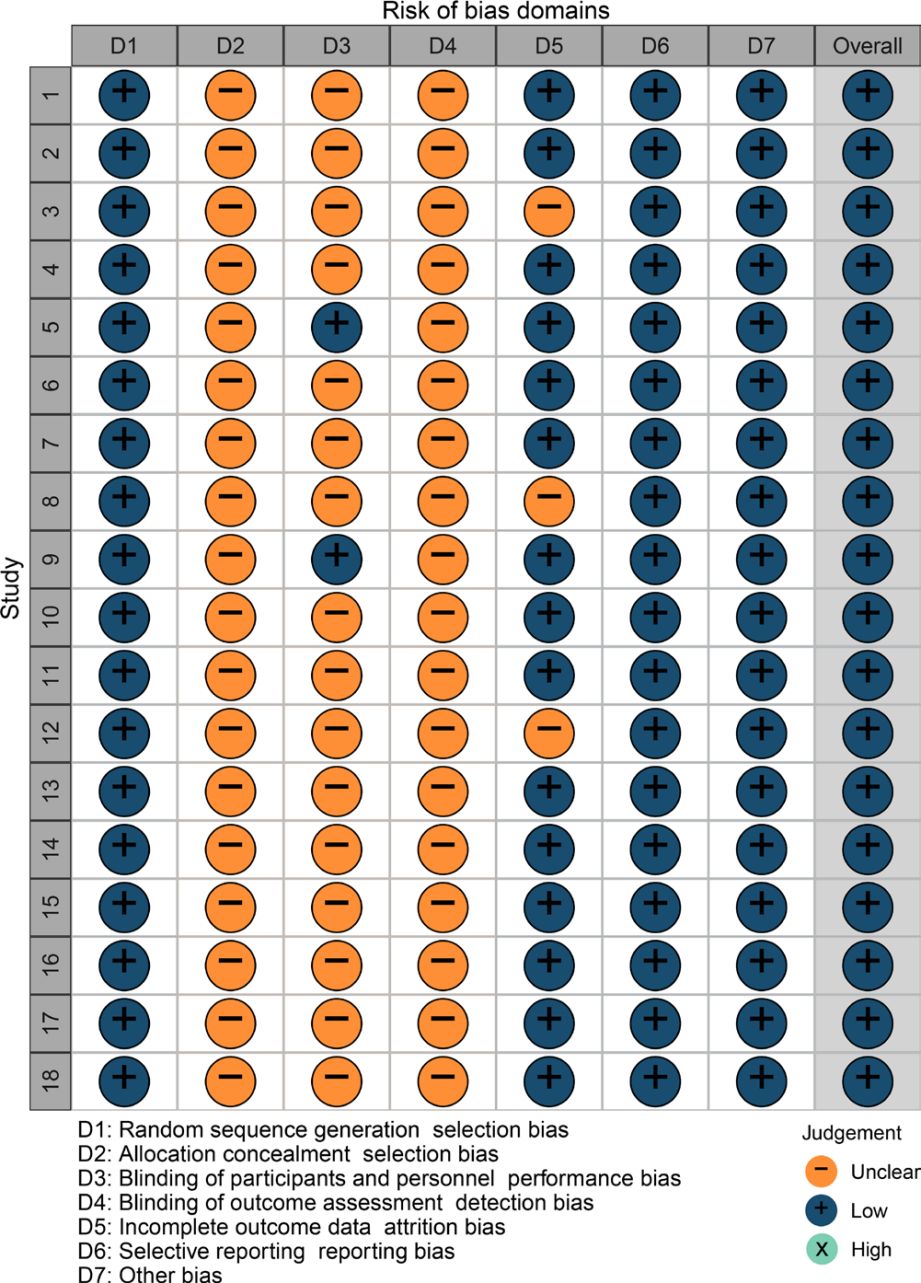
Risk-of-bias summary.

A total of 1093 patients were enrolled in this study, which consisted of 18 randomized controlled trials. Among them, 550 patients were assigned to the treatment group, while 543 patients were assigned to the control group. The baseline data comparison between the treatment and control groups in the included studies demonstrated no significant differences (*P* > .05). Moreover, the studies exhibited clear inclusion and exclusion criteria. In terms of overall study quality assessment, 7 studies provided descriptions of specific randomization methods, all of which utilized the random number table method.^[15,16,19,21,28–30]^ However, none of the studies reported whether allocation concealment was implemented. Only 2 studies mentioned the use of a blinded method,^[20,24]^ and 3 studies documented sample loss and dislodgement.^[18,23,27]^ The risk of study bias is visually presented in Figures 2 and 3.

### 3.3. Meta-analysis

#### 3.3.1. Effects of Fuzheng–Buyi formula on total effective rate

**Figure 4.**
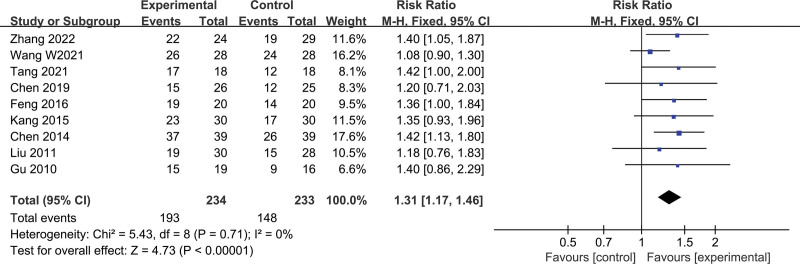
Forest plot of total effective rate.

A total of 9 studies with 467 study subjects (234 cases in the treatment group and 233 cases in the control group) were included, with no statistical heterogeneity between studies (*I*^2^ = 0%, *P* = .71), using a fixed-effect model. The results showed that the total effective rate of the Chinese herbal compound combined with Western medicine compared with Western medicine alone for CRPC was statistically significant (RR = 1.31, 95% CI [1.17, 1.46], *P* < .00001), and the effective rate in the combined medicine group was more significant (Fig. 4).

#### 3.3.2. Adverse effects of Fuzheng–Buyi formula on total effective rate

**Figure 5.**
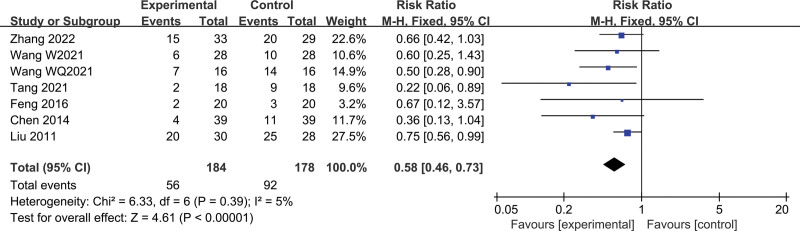
Forest map of adverse reaction incidence.

Adverse effects: a total of 7 studies were included, including 362 subjects (184 cases in the treatment group and 178 cases in the control group). There was no statistical heterogeneity between studies (*I*^2^ = 5%, *P* = .39), using a fixed-effect model. The results revealed a lower incidence of adverse reactions with the Chinese herbal compound combined with Western medicine compared to Western medicine alone in patients with CRPC (RR = 0.58, 95% CI [0.46, 0.73], *P* < .00001) (Fig. 5).

#### 3.3.3. Effects of Fuzheng–Buyi formula on TCM symptom score

**Figure 6.**
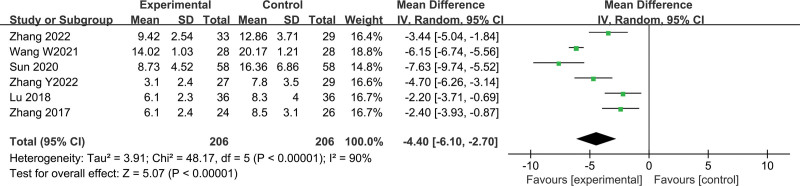
Forest plot of TCM symptom score. TCM = Traditional Chinese Medicine.

A total of 6 studies with 412 study subjects (206 in the treatment group and 206 in the control group) were included, with heterogeneity between studies (*I*^2^ = 90%, *P* < .00001), and the results did not change significantly after sensitivity analysis, using a random-effects model. The results showed that the combination of the Fuzheng–Buyi method with Western medicine significantly reduced the TCM symptom score of patients compared to Western medicine alone (MD = −4.40, 95% CI [−6.10, −2.70], *P* < .00001) (Fig. 6).

#### 3.3.4. Effects of Fuzheng–Buyi formula on quality of life

**Figure 7.**
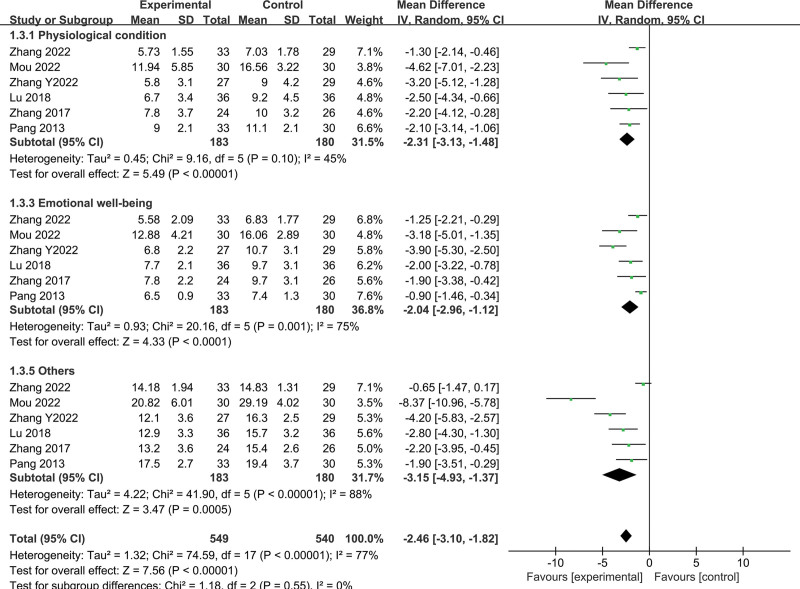
Forest map of FACT-P score after sensitivity analysis (adverse factors). FACT-P = functional assessment of cancer therapy-prostate.

**Figure 8.**
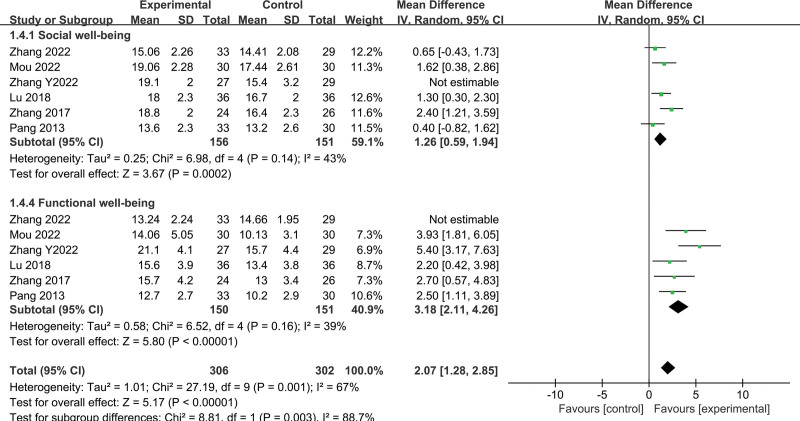
Forest map of FACT-P score after sensitivity analysis (favorable factors). FACT-P = functional assessment of cancer therapy-prostate.

A total of 6 studies comprising of 363 subjects (183 in treatment group and 180 in control group) evaluated the quality of life using functional assessment of cancer therapy-prostate scores. The analysis revealed the following (Figs. 7 and 8):

① Physiological condition: the random-effects model showed statistical heterogeneity (*I*^2^ = 45%, *P* = .1), with the combination of the Fuzheng–Buyi and Western medicine being better than Western medicine alone in improving the physiological condition of CRPC patients (MD = −2.31, 95% CI [−3.13, −1.48], *P* < .00001). ② Social well-being: the random-effects model showed statistical heterogeneity (*I*^2^ = 71%, *P* = .004), and after sensitivity analysis by excluding 1 study, the combination of the Fuzheng–Buyi with Western medicine was found to be better in improving the social well-being of CRPC patients than the control group (MD = 1.26, 95% CI [0.59, 1.94], *P* = .0002). ③ Emotional well-being: the analysis showed statistical heterogeneity between studies (*I*^2^ = 75%, *P* = .001), and the positive effect of the Fuzheng–Buyi combined with Western medicine on the emotional status of CRPC patients was found to be better than the control group (MD = −2.04, 95% CI [−2.96, −1.12], *P* < .0001). ④ Functional well-being: there was statistical heterogeneity between studies (*I*^2^ = 90%, *P* < .00001), and the combination of the Fuzheng–Buyi with Western medicine was better than Western medicine alone in restoring the functional well-being of CRPC patients (MD = 3.18, 95% CI [2.11, 4.26], *P* < .00001). ⑤ Additional concern: there was statistical heterogeneity between studies (*I*^2^ = 88%, *P* < .00001), and the Fuzheng–Buyi method combined with Western medicine was found to be more effective in treating patients with CRPC (MD = -3.15, 95% CI [−4.93, −1.37], *P* = .0005).

#### 3.3.5. PSA levels of Fuzheng–Buyi formula on quality of life

**Figure 9.**
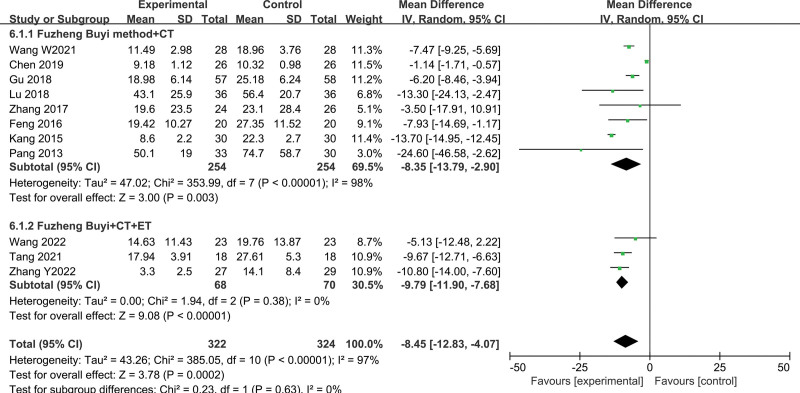
Forest plot of PSA levels. PSA = prostate-specific antigen.

PSA levels: 11 studies comprising of 646 cases (322 in treatment group and 324 in control group) showed heterogeneity between studies, which may be attributed to variations in specific interventions. Among these studies, 8 utilized ET,^[19,23–28,30]^ 2 employed a combination of ET and CT,^[15,17]^ and 1 used CT.^[21]^ Since only 1 study involved CT and there was overlap between the intervention methods, it was grouped with the ET combined with CT. Specific subgroup analyses were conducted based on differences in intervention methods among the studies. After combining the results, significant heterogeneity remained in the ET group (*I*^2^ = 98%, *P* < .00001), while there was no heterogeneity in the ET plus CT/CT group (*I*^2^ = 0%, *P* = .38). Considering potential sources of heterogeneity, a random-effects model was used for analysis. The results showed that in the endocrine subgroup, the combination of Fuzheng–Buyi with Western medicine was significantly more effective than Western medicine alone in reducing PSA levels in patients with CRPC (MD = −8.35, 95% CI [−13.79, −2.90], *P* = .003). In the ET plus CT/CT subgroup, the combination group was also more effective in reducing PSA levels in patients with CRPC than the control group (MD = −9.79, 95% CI [−11.90, −7.68], *P* < .00001). The analysis revealed a significant reduction in PSA levels in CRPC patients with the combination of Fuzheng–Buyi with Western medicine compared to Western medicine alone (Fig. 9).

### 3.4. Publication bias

**Figure 10.**
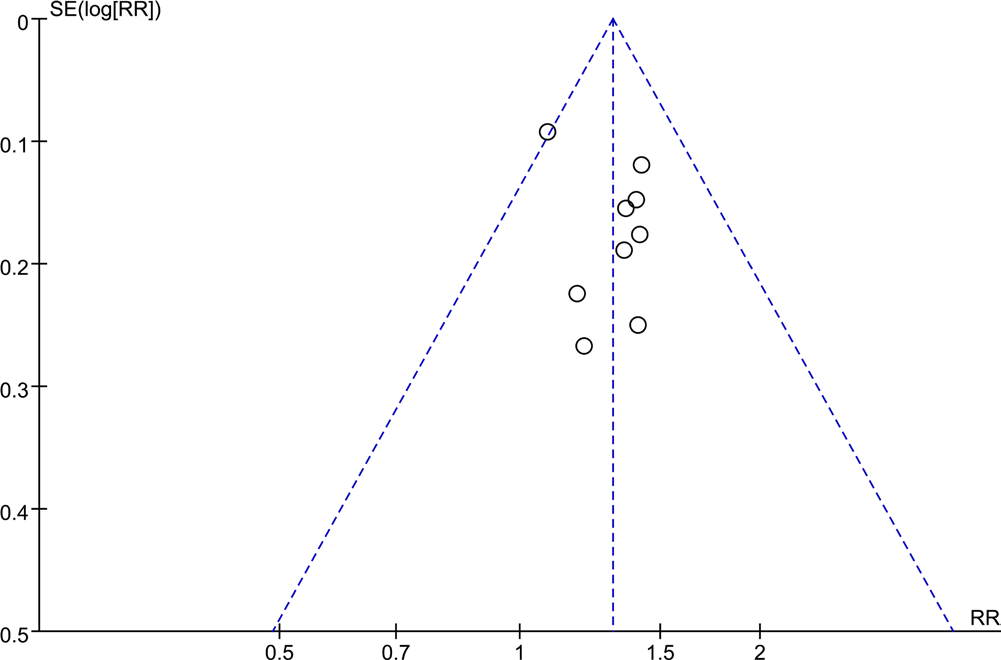
Funnel plot of total effective rate.

A funnel plot was constructed using the total effective rate as the primary outcome measure. The funnel plot appears asymmetric, indicating that the possibility of publication bias is nonneglegible (Fig. 10).

## 4. Discussion

According to the Cochrane quality assessment criteria, a total of 19 studies were included in this analysis. No survival analysis was conducted due to missing follow-up data. The meta-analysis results showed that Chinese herbal compound combined with Western medicine was more effective in terms of overall efficiency, TCM evidence score, quality of survival, and control of PSA levels for CRPC patients compared to Western medicine alone. Furthermore, the combined treatment approach resulted in a lower incidence of adverse events.

It is important to note that CRPC is a concept rooted in Western medical theory and treatment, and there is no direct equivalent term in Chinese medicine. In TCM theory, the general pathogenesis of CRPC involves a combination of deficiency and excess, primarily attributed to an imbalance of yin and yang, insufficiency of kidney qi, deficiency of kidney yang, and depletion of yin essence.^[33]^ Treatment strategies based on the principles of tonifying kidney deficiency and eliminating pathogenic factors are recommended.^[34,35]^ The study identified that Chinese herbal formulas utilized in the treatment of CRPC consist of Chinese herbs with properties of supporting the righteousness, strengthening the spleen, benefiting the kidney, and resolving blood stasis. Some examples of these herbs include prepared liquorice root, tangshen, milkvetch root, malaytea scurfpea fruit, and solomonseal rhizome. Based on individual patient conditions, modifications can be made to achieve the purpose of tonifying the kidneys, nourishing the righteous energy, and eliminating pathogenic factors.

In this systematic review and meta-analysis, results showed that the therapeutic efficacy of the combination of Fuzheng–Buyi formula and Western medicine was better than that of the controls, but some of the randomized methods included in the study were unclear, such as no allocation concealment and blindness used, limited number of samples, and no sample number estimated in all studies. Furthermore, Chinese medicine formulas are typically individualized based on symptom identification and treatment, resulting in variations in the composition of prescriptions for different patients. This makes it challenging to quantitatively compare the specific effects of individual herbs or drugs on efficacy, thus increasing the instability of the results. Collectively, in order to propose a convincing conclusion in this topic, further multi-center randomized and double-blind controlled trials are still needed.

## 5. Conclusions

Taken together, this meta-analysis reveals that combination of Fuzheng–Buyi formula and Western medicine was more effective in improving the clinical efficacy and quality of life of CRPC patients, with lower incidence of adverse events compared with Western medicine alone. However, whether the therapeutic efficacy of Fuzheng–Buyi formula alone is better than Western medicine treatments is uncertain, and a rigorous, large-sample randomized double-blind controlled trial is needed.

## Author contributions

**Data curation:** Shirong Peng.

**Methodology:** Jia Xu.

**Supervision:** Wenjuan Liu.

**Software:** Hailong Wang.

**Visualization:** Xiao Liang.

**Writing – original draft:** Xiao Liang.

**Writing – review & editing:** Hai Huang, Qi Li.
